# Age-Related Cellular Copper Dynamics in the Fungal Ageing Model *Podospora anserina* and in Ageing Human Fibroblasts

**DOI:** 10.1371/journal.pone.0004919

**Published:** 2009-03-23

**Authors:** Christian Q. Scheckhuber, Jürgen Grief, Emmanuelle Boilan, Karin Luce, Florence Debacq-Chainiaux, Claudia Rittmeyer, Ricardo Gredilla, Bernd O. Kolbesen, Olivier Toussaint, Heinz D. Osiewacz

**Affiliations:** 1 Institute of Molecular Biosciences, Johann Wolfgang Goethe University, Frankfurt am Main, Germany; 2 Research Unit on Cellular Biology, University of Namur, Namur, Belgium; 3 Institute of Inorganic Chemistry/Analytical Chemistry, Johann Wolfgang Goethe University, Frankfurt am Main, Germany; Ordway Research Institute, United States of America

## Abstract

In previous investigations an impact of cellular copper homeostasis on ageing of the ascomycete *Podospora anserina* has been demonstrated. Here we provide new data indicating that mitochondria play a major role in this process. Determination of copper in the cytosolic fraction using total reflection X-ray fluorescence spectroscopy analysis and *eGfp* reporter gene studies indicate an age-related increase of cytosolic copper levels. We show that components of the mitochondrial matrix (i.e. eGFP targeted to mitochondria) become released from the organelle during ageing. Decreasing the accessibility of mitochondrial copper in *P. anserina* via targeting a copper metallothionein to the mitochondrial matrix was found to result in a switch from a copper-dependent cytochrome-c oxidase to a copper-independent alternative oxidase type of respiration and results in lifespan extension. In addition, we demonstrate that increased copper concentrations in the culture medium lead to the appearance of senescence biomarkers in human diploid fibroblasts (HDFs). Significantly, expression of copper-regulated genes is induced during *in vitro* ageing in medium devoid of excess copper suggesting that cytosolic copper levels also increase during senescence of HDFs. These data suggest that the identified molecular pathway of age-dependent copper dynamics may not be restricted to *P. anserina* but may be conserved from lower eukaryotes to humans.

## Introduction

During the course of investigations to unravel the molecular mechanisms leading to an increased lifespan in the nuclear long-lived grisea mutant of the fungal ageing model *Podospora anserina*, an impact of a tight regulation of cellular copper levels was identified [1]–[5]. It was shown that all processes leading to cellular copper depletion result in cytochrome-c oxidase (COX) deficiency due to the dependence of this respiratory chain complex on copper as a cofactor. As a retrograde response, copper depleted strains induce an alternative terminal oxidase (AOX) which utilizes iron as a cofactor. In the grisea mutant, the generation of ROS at the inner mitochondrial membrane was demonstrated to be lower than in the wild-type strain [6]. Apart from COX, another copper-dependent enzyme, Cu/Zn superoxide dismutase (SOD1), was demonstrated to influence lifespan of *P. anserina* cultures [3] also by its impact on cellular ROS levels. While respiration strongly affects the generation of ROS [6], [7], the latter system is involved in the degradation of these harmful compounds. During the course of investigations, data were obtained suggesting age-related increases in cytosolic copper levels during ageing of *P. anserina* cultures. This conclusion was drawn from transcript levels of the copper regulated genes *PaMt1*, *PaCtr3*, *PaSod2* which were found to be either increased or reduced in senescent fungal cultures [2], [5]. In addition PaSOD1 activity, depending on copper as a cofactor, was found to be higher in senescent cultures, while PaSOD2 activity, as a result of reduced transcript levels, is decreased.

On the basis of these findings and the fact that mitochondrial activity decreases during ageing, a possible age-related release of copper from mitochondria was suggested to be responsible for increased copper levels in the cytoplasm of senescent wild-type cultures [2], [3], [5]. This possibility became even more attractive when a non-proteinaceous copper pool was demonstrated to exist in the mitochondrial matrix of yeast and mammalian cells [8], [9], that was suggested to be utilized for metallation of COX and SOD1 in the mitochondrial intermembrane space [9].

Here we report data of an analysis measuring copper content in mitochondria and the cytosolic fraction of juvenile vs. senescent *P. anserina* strains. In the senescent mycelia, cytosolic copper levels are strongly increased. We also show that eGFP targeted to the mitochondrial matrix becomes released from mitochondria during senescence. Moreover, we analyzed the consequences of targeting a copper binding non-mitochondrial protein (PaMT1) to the mitochondrial matrix on the type of respiration and on lifespan. Finally, we provide data indicating that increased copper levels can induce biomarkers of senescence and that genes known to be regulated by copper in humans also show senescence-related overexpression in replicatively senescent and stress-induced senescent human diploid fibroblasts (HDFs), another extensively investigated experimental ageing model [10]–[12]. These data suggest that the identified molecular pathway may not be restricted to *P. anserina* but may be conserved from lower eukaryotes to humans.

## Materials and Methods

### P. anserina strains, cultivation and transformation

For all experiments, unless otherwise noted, *Podospora anserina* wild-type strain s [13] was used, grown on standard cornmeal medium under standard conditions [14]. Copper deprivation of *P. anserina* mycelia was achieved by supplementation of cornmeal agar with 33 µM BCS (Bathocuproinedisulphonic acid, Sigma-Aldrich, USA) and 1 mM ascorbic acid.

*P. anserina* protoplasts were transformed according to the previously published protocols [15], [16] except that 1×10^7^ protoplasts were used instead of 1×10^8^. For selection, hygromycin B (100 µg/ml) was added to the transformation medium.

Lifespan of *P. anserina* was determined in race tubes as previously described [16].

### Plasmid constructs

To direct proteins (PaMT1 or eGFP) into the matrix of mitochondria, a mitochondrial targeting sequence (MTS) had to be cloned. As no distinct MTS of *P. anserina* was available, the MTS of the mitochondrial processing peptidase (MPP) of the close relative *Neurospora crassa* was used (first 35 amino acids of the pre-protein) [17]. The sequence was amplified via PCR using the primers NcMPP MTS for (5′ ccggatccgatggtcaatctaggaagc 3′ - adding a 5′ *Bam*HI-site) and NcMPP MTS rev (5′ attaagcttcgccatggcgaggccggccatcgt 3′ - adding an in-frame *Nco*I-site and a *Hin*dIII-site at the 3′ end) and genomic DNA of a *N. crassa* wild-type (kindly provided by H. Bertrand, Michigan State University). The PCR product was cloned via *Bam*HI and *Hin*dIII into pEX-ble, a pUC19 derivative containing a 560 bp fragment of the promoter of *PaMt1*, leading to strong constitutive expression, resulting in pEX-NcMTS. The 3′-tagged *PaMt1* was amplified by PCR using primers PaMT1 for (5′ aaccatgggtggctcttgcaac 3′ - adding a 5′ *Nco*I-site) and PaMT1-6×His rev (5′ ccttaatggtgatggtggtgatgtttgcatccagagcaggagcctatag 3′) from genomic DNA of the *P. anserina* wild-type strain s. This PCR product was cloned into pMOS (Amersham Biosciences). The 811 bp fragment containing the transcriptional terminator of *PaMt1* (*PaMt1*-TT) was PCR-amplified using primers PaMT1 TT for (5′ ggtctagagttattccctcactcaca 3′) and *PaMT1 TT rev* (5′ ggaagcttacatttgatcct 3′) and inserted into *Xba*I, *Hin*dIII site behind the tagged *PaMt1* reading frame. Subsequently, an *Nco*I, *Hin*dIII fragment containing the tagged *PaMt1* and *PaMt1*-TT was excised from the resulting plasmid and ligated into pEX-NcMTS. The final expression plasmid was termed pEX-NcMTS-PaMT1-His. For selection in *P. anserina* the hygromycin B-resistance-cassette from pSM1 [18] was inserted via the *Eco*RI site. For directing eGFP into the mitochondrial matrix, the PaMT1-ORF of pEX-NcMTS-PaMT1-His was replaced by the *eGfp*-coding sequence from pSM1 [18] via the *Nco*I and *Xba*I sites.

For construction of a copper sensor plasmid, a 4.3 kbp *PaMt1* promoter fragment was amplified using primers PMT-XbaI-for (5′ tggcggccgctctagaggagttgaagagtaatcc 3′) and PMT-PstI-rev (5′ ttgatatcgaattcctgcagtttgatagttgtttgggg 3′) from cosmid CPs 24A3 of a genomic library of the *P. anserina* wild-type strain s. The PCR product was restricted using *Xba*I/*Pst*I and cloned into the *Xba*I/*Pst*I site of plasmid pSM4 which is a derivative of EGFP reporter plasmid pSM2 [18]. The resulting plasmid, which lost the *eGfp*-Gene in the previous cloning step, was termed pSM4-P*Mt*. To re-insert *eGfp* into the construct, a PCR fragment containing the gene was amplified using primers PstI-EGFP-f (5′ aactgcagatggtgagcaagggcgagg 3′) and EcoRV-EGFP-r (5′ aagatatccggccgctctagaaagaagg 3′) from plasmid pSM4. After restriction using *Pst*I and *Eco*RV the PCR fragment was cloned into the *Pst*I/*Eco*RV site of pSM4-P*Mt*. The resulting plasmid was termed pSM4-P*Mt*-EGFP.

### Isolation of mitochondria and cytosol

For protein localization experiments and O_2_-consumption measurements, mitochondria were isolated by differential centrifugation as described earlier [6].

For TXRF-measurements and isolation of mtDNA the following changes and additions were applied: the composition of the buffer was changed to 0.6 M sorbitol, 10 mM Tris/Acetate, 1 mM EDTA, 0.2% BSA (pH 7.5). The supernatant after the first high-speed centrifugation step (15,000 g) (‘post-mitochondrial supernatant’) was designated as ‘cytosol’. Western-blot experiments revealed that this fraction does not contain substantial mitochondrial protein contaminations. To remove all traces of contamination by vacuoles in the mitochondrial fraction, the final pellet of the differential centrifugation was loaded on a 20%/36%/50% discontinuous sucrose gradient and subjected to ultracentrifugation (100,000 g, 4°C, 1 h in a Sorvall Ultra Pro 80 with a TH 641 rotor). The band at the 36%/50% interface was collected and centrifuged in isolation buffer without BSA (20 min, 4°C, 15,000 g). The pellet was resuspended in an appropriate volume of isolation buffer without BSA and the protein concentration was determined according to [19].

### Total reflection X-ray fluorescence (TXRF) measurements

TXRF is a well established method for the quantitative determination of metals in bio-organic matrices like proteins and enzymes [20], [21]. With typical amounts of protein and enzyme samples in the µM range, amounts of metals in the sub-nanogram order can be determined. In this work the measurements of copper content were performed on an EXTRA II A spectrometer (Atomika Instruments, Oberschleissheim, Germany) with MoK α-radiation, a measuring time of 1000 s and a sample volume of 3 µl. As an internal standard 1 µl of a 1 mg/l Rb standard solution was added. 4 µl of sample+standard were pipetted on a plane glass quartz carrier and the solvent was vaporized under clean bench ambient [21]. All measurements were done in quadruplicate.

### Oxygen consumption measurements

To characterize the type of terminal oxidase used in the respiratory chain, the consumption of oxygen and its sensitivity to different inhibitors was measured on freshly isolated mitochondria as described previously [6]. The final concentrations of inhibitors were: 10 mM KCN (COX inhibitor) and 4 mM SHAM (salicyl hydroxamic acid, AOX inhibitor).

### Detection of reorganized mitochondrial DNA

In *P. anserina*, a well established marker for senescence is the age-related amplification of so-called ‘plasmid-like DNA’ (plDNA) and the reorganization of mitochondrial DNA (mtDNA) [22], [23]. For isolation of the mtDNA, 1 mg of isolated mitochondria were treated with 0.5 mg/ml Proteinase K for 10 min, following a phenol∶ chloroform∶ isoamylalcohol (25∶24∶1) extraction and ethanol precipitation of the DNA. Equal amounts of DNA were digested with *Bgl*II, gel-fractionated, transferred to a Hybond-N membrane (Amersham) and hybridized with a plDNA-specific probe (generated from plasmid pSP17 [24]).

### Western Blot analysis of P. anserina proteins

Mitochondrial preparations were separated on standard 12% glycine SDS polyacrylamid gels. Due to the small size of the His-tagged PaMT1, mitochondrial proteins of PaMT1-his-transformants were separated on 12% tricine SDS-polyacrylamid-gels. Following electrophoresis proteins were transferred to PVDF membranes (Millipore). The following antibodies were used: mouse α-AOX (raised against the AOX of *Sauromatum guttatum*) [25], rabbit α-Cu/Zn-SOD (Stressgen), mouse α-GFP (Molecular Probes), mouse α-His (Santa Cruz), rabbit α-MnSOD (Stressgen) and rabbit α-Porin (gift from the group of T. Langer, University of Cologne, Germany). Binding of antibodies was visualized using HRP-coupled secondary antibodies (goat α-mouse and goat α-rabbit – both from Sigma) and the ‘Western Blotting Luminol Reagent’ (Santa Cruz Biotechnology). To reprobe the membranes, blots were stripped in glycine stripping buffer (0.2 M glycine, 1% Tween 20, 0.1% SDS, pH 2.2) followed by extensive washing in PBS.

### P. anserina in situ immunodetection

*P. anserina* mycelia were grown on glass slides which central depressions contained a 1∶1 mixture of cornmeal agar and 2% agarose for two days in a wet chamber at 27°C. For fixation, mycelia were treated with 70% ethanol (ice-cold) for 5 min at RT. After washing the culture (rinsing with sterile water), the cell walls of the mycelia were partially degraded by treatment with the enzyme-mixture Glucanex (Novo Nordisk Ferment AG, 20 mg/ml in TPS buffer [5 mM Na_2_HPO_4_×2 H_2_O, 45 mM KH_2_PO_4_, 0.58 M sucrose, pH 5.5 ]) for 30 min at 35°C. Following washing, mycelia were overlaid with a 1% Triton X-100 (in TPS) solution for 30 min at RT. After a second round of washing the isolates were overlaid with the primary antibody solution (1/150 α-His [Santa Cruz] in TPS buffer containing 10% PEG stock solution [60% PEG 4000, 50 mM CaCl_2_ * 2 H_2_O, 10 mM Tris/HCl pH 7.5]) overnight at 4°C. The samples were washed and incubated with the secondary antibody solution (1/2000 mouse α-IgG coupled with the Alexa Fluor 488 nm dye [Invitrogen] in TPS buffer containing 10% PEG stock solution) for three hours in the dark at RT. Following a final washing step, mycelia were analyzed using a DM LB fluorescence microscope (Leica, Wetzlar, Germany) equipped with the appropriate filters (excitation 450–490 nm/emission >515 nm).

### Transcript analysis of P. anserina genes by semi quantitative RT-PCR

Isolation of RNA and semi quantitative RT-PCR of *P. anserina* genes has been described elsewhere [2], [6]. The utilized primers were: PaMT1 for (5′ aaccatgggtggctcttgcaac 3′), PaMT1 rev (5′ ccttatttgcatccagagcagga 3′), PaPorin 8 (5′ gtctcggtgcctctttcg 3′), PaPorin 9 (5′ tgtttgccctgatcatcg 3′).

### Fluorescence microscopy

For investigating the release of eGFP from senescent mitochondria, a piece of transgenic mycelium was inoculated on a small block of cornmeal medium and covered with a cover slip. After two days of incubation at 27°C in the light, hyphae grew on the cover slip. These were subjected to microscopic analyses, performed with a DM LB fluorescence microscope (Leica, Wetzlar, Germany) using the filter [excitation 470/40 nm/emission 525/50 nm] for detection of eGFP. For staining of mitochondria, MitoTracker® red CMXRos was added in a concentration of 0.66 µM and the samples were directly examined without prior incubation. For analyzing the copper dependent activation of *eGfp* expression, a piece of transgenic mycelium was squeezed between object carrier and cover slip and subsequently analyzed microscopically. Pictures were taken with the DC 500 camera (Leica, Wetzlar, Germany).

### Cell culture and induction of premature senescence

AG04431 Skin HDFs (FS) (Coriell Institute for Medical Research, USA) and WI-38 fetal lung HDFs (AG06814, Coriell Cell Repositories, USA) were grown in BME medium (Invitrogen, UK)+10% (v∶v) of fetal calf serum (FCS) (Invitrogen, UK) and 2 mM L-glutamine. The medium was changed every four days in slowly growing cultures. Cells unable to make a population doubling within two weeks were considered as replicatively senescent.

To initiate stress-induced premature senescence (SIPS), AG04431 skin HDFs (FS) were subcultivated at half confluence (10,000 cells/cm^2^) in BME+1% FCS at 55–60% of their *in vitro* proliferative life span. At 72 hours after plating, the cells were washed once with phosphate buffer saline (10 mM phosphate, 155 mM NaCl, pH 7.4) (PBS) and exposed to UVB radiation (250 mJ/cm^2^) in a thin layer of PBS using three Philips TL 20W/01 lamps (Philips, The Netherlands) emitting UVB peaking at 311 nm and placed at 30 cm above the flaks. The emitted radiation was checked under flask lid using an UVR-radiometer with a UVB sensor (Bioblock Scientific, Belgium). After radiation, PBS was replaced by BME+1% FCS. UVB stress was performed twice a day for five days [11]. Control cells were exposed to the same culture conditions without UVB exposure.

### Incubation of cells with copper and sodium salts

To analyze the impact of copper, WI-38 fetal lung HDFs were subcultivated at half confluence (20,000 cells/cm^2^) in BME+10% FCS at 55–60% of their *in vitro* proliferative life span. At 24 hours after plating, the medium was changed with new medium completed with 500 µM of copper sulfate (CuSO_4_), copper chloride (CuCl_2_), sodium sulfate (Na_2_SO_4_) or sodium chloride (NaCl). The cells were incubated for 16 hours. After incubation, the cells were washed once by phosphate buffer saline (10 mM phosphate, 155 mM NaCl, pH 7.4) (PBS). PBS was replaced by BME+10% FCS. Control cells were exposed to the same culture conditions without copper or sodium salts.

### Detection of copper by electron microscopy

After incubation, the cells were washed once by phosphate buffer saline (10 mM phosphate, 155 mM NaCl, pH 7.4) (PBS) and were fixed using 2.5% glutaraldehyde in cacodylate buffer (0.2 M, pH 7.4) for 2.5 h. The cells were washed three times in cacodylate buffer and were post-fixed with a solution of 1% osmium tetroxide (OsO_4_) in cacodylate buffer. The samples were maintained in 0.2 M cacodylate buffer overnight at 4°C. The samples were dehydrated by different washing steps in ethanol 30°, 50°, 70°, 85° and 100° (once for 5 min and once for 10 min in each one). The samples were washed four times 5 min with propylene oxide and twice 15 min with propylene oxide/resin (v∶v) with agitation. The samples were incubated with resin overnight at 37°C, 24 hours at 45°C and 72 hours at 60°C and were prepared for electron microscopy. Transmission electron microscopy (TEM) images were taken using a JEOL 7500F transmission electron microscope operating at an acceleration voltage of 15 kV. The JEOL 7500F microscope, allowing observation of granules with a resolution of 1.6 nm, is also equipped with an Energy Dispersive X-ray (EDX) detector for the analysis of the elementary composition (qualitative and quantitative).

### Transcript analysis of HDF genes by real time PCR

At 72 hours after the last UVB-stress or at 24 hours after the end of incubation with copper salts/sodium salts or when the cells were senescent, total RNA was extracted from three or two independent HDF cultures using ‘Total RNAgent’ extraction kit (Promega, USA). Total RNA (2 µg) was reversed transcribed using SuperScript II Reverse Transcriptase (Invitrogen, UK). Primers (Table 1) were designed using the ‘Primer Express 1.5 software (Applied Biosystems, The Netherlands). Amplification reactions assays contained 1×SYBR Green PCR Mastermix and primers (Applied Biosystems, The Netherlands) at optimal concentration. A hot start at 95°C for 5 minutes was followed by 40 cycles at 95°C for 15 seconds and 65°C for 1 minute using the 7000 SDS thermal cycler (Applied Biosystems, The Netherlands). Melting curves were generated after amplification and data were analyzed using the thermal cycler software. Each sample was tested in triplicate or duplicate.

**Table 1:** Forward (F) and Reverse (R) primers used for real-time PCR.

Primer name	Sequence 5′→3′	Length	Amplicon size	Accession no.	Reference
prion protein-713F	atgatggagcgcgtggtt	18	84 bp	NM_000311	[78]
prion protein-796R	catgctcgatcctctctggtaa	22			
HSPA1A-1959F	gagaaggacgagtttgagcacaa	23	74 bp	NM_005345	[79]
HSPA1A-2032R	tggtacagtccgctgatgatg	21			
MT2A-238F	gcctgatgctgggacagc	18	88 bp	NM_005953	[80]
MT2A-325R	ggtcacggtcagggttgtacata	23			
p21-495F	ctggagactctcagggtcgaa	21	123 bp	NM_000389	[81]
p21-617R	ccaggactgcaggcttcct	19			
GAPDH 942-963F	acccactcctccacctttgac	21	111bp	NM_002046	[82]
GAPDH 1033-1053R	gtccaccaccctgttgctgta	21			
RPL13A-387F	ctcaaggtcgtgcgtctgaa	20	94 bp	NM_012423	
RPL13A-480R	tggctgtcactgcctggtact	21			

### Western blot analysis of HDF proteins

Cells were washed once with ice-cold PBS and lysed in ice-cold lysis buffer DLA (Urea 7 M, Thiourea 2 M, Chaps 2%, DTT 2%). The cells were agitated, sonicated and centrifuged at 13,000 g for 10 min. The supernatants were stored. Proteins were assayed using the Bradford reagent (Bio-Rad) before electrophoresis (10% SDS-PAGE), transferred to a polyvinylidene difluoride membrane (Amersham Biosciences) and probed with the following antibodies: α-Hsp70 goat IgG ((K20) – Santa Cruz Biotechnology, USA), α-PrP rabbit IgG ((EP1802Y) – Abcam, UK), α-α-tubulin mouse IgG (Clone B-5-1-2 – Sigma-Aldrich, USA) and horseradish peroxidase-linked secondary antibodies (α-rabbit and α-mouse antibodies – Amersham Biosciences; α-goat antibodies – DakoCytomation, Glostrup, Denmark). α-tubulin was used as a reference protein check equal loading on the gels. The bands were visualized after incubation with chemiluminescent substrates (ECL detection kit – Amersham Biosciences). Scanning was performed with the Image Master Labscan version 2003.01 software (Amersham Biosciences).

### Senescence biomarkers analysis: cytochemical staining of SA β-galactosidase activity

At 48 hours after incubation, cells were seeded in 6-well culture plates (Corning) at a density of 10,000 cells/well. Senescence-associated (SA) β-galactosidase activity was determined 24 h later as described by [26]. The amount of SA β-gal positive cells is given as the percentage of the total number of cells counted in each well. Each result is the mean value obtained by counting 400 cells on three independent culture dishes±SD. Statistical analysis was carried out with the Student's *t*-test.

### Measurement of DNA synthesis

Cells were seeded in 24-well culture plates (Corning) at a density of 10,000 cells/well and grown in 1 ml of BME+10% FCS supplemented with 1 µCi [^3^H]- thymidine (specific activity: 2 Ci/mmol; DuPont, NEN, USA) during 24 h. The cells were then washed twice with 1 ml PBS, fixed with 1 ml of ice-cold 10% trichloroacetic acid (TCA), washed once with 1 ml 70% ethanol and once more with 1 ml of PBS. About 250 µl of 0.5 M of NaOH was added for 30 min and neutralized with 250 µl of 0.5 M HCl. The incorporated radioactivity was quantified by a scintillation counter (Packard Instrument Company, USA). Data were normalized to the cellular protein content assayed by the Folin method [27]. The results are expressed as mean of triplicates±SD. Statistical analysis was carried out with the Student's *t*-test.

## Results

### Age-related changes of copper levels in the cytoplasm

Previous studies suggested an increase in cytosolic copper levels during ageing of *P. anserina* cultures. This conclusion was drawn from transcript analyses of copper-regulated genes and from SOD activity analyses [2]–[5]. Copper increase in the cytosolic cell fraction was hypothesized to result from a release of the metal from mitochondria [4]. In the current study we investigated the age-related changes in copper abundance more directly by measuring copper concentrations in the post-mitochondrial (cytosolic) supernatant and in mitochondria purified by sucrose-gradient density centrifugation. Copper was measured by total reflection X-ray fluorescence spectroscopy (TXRF) in samples prepared from juvenile and senescent *P. anserina* cultures.

**Figure 1:**
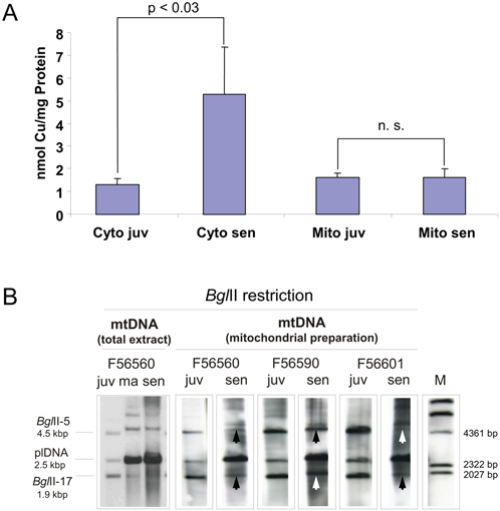
Increase in cytoplasmic copper concentration during ageing of *P. anserina* wild-type s. (A) Cellular extracts were fractionated by differential centrifugation and subsequent sucrose-gradient ultra-centrifugation and the copper content of cytosol and mitochondria was determined by TXRF. The values represent mean±SE. Statistical analysis: Wilcoxon test, n.s.: nonsignificant (p>0.05). (B) DNA preparations were hybridized to a plDNA-specific probe after digestion with *Bgl*II and transferred onto a nylon membrane. In juvenile strains signals of 4.5 kbp (*Bgl*II-4) and 1.9 kbp (*Bgl*II-17), indicative for intact mtDNA, are detected which are replaced by the signal of amplified plDNA (2.5 kbp) in senescent preparations. In the mtDNAs isolated from mitochondria of senescent strains, a greater amount of 4.5 kbp and 1.9 kbp fragments is observed (arrows) compared to the analysis which was performed using a total DNA preparation from a senescent strain including nuclear DNA and mtDNA. M: DIG-labelled λ DNA [*Hin*dIII].

As expected, a clear increase in copper levels was observed in the post-mitochondrial fraction isolated from senescent cultures (juv: 1.3±0.3 nmol Cu/mg protein; sen: 5.3±2.1 nmol Cu/mg protein; p<0.03, Wilcoxon test, two-tailed) (Fig. 1A). However, in contrast to our expectation, copper concentrations in the mitochondrial fraction did not change significantly. We therefore verified the ‘age’ of the isolated mitochondria investigating the reorganization of mitochondrial DNA (mtDNA) in the cytochrome-c oxidase subunit I (*CoxI*) gene region. Rearrangements occurring in this region are a robust biomarker of ageing in *P. anserina* [23], [28]. The analysis revealed that in mitochondrial fractions purified by sucrose gradients a significantly higher fraction of two mtDNA *Bgl*II fragments, 1.9 kbp and 4.5 kbp in size, are present in senescent cultures than in total DNA preparations. In addition, plDNA, a 2.5 kbp autonomous DNA molecule liberated from the corresponding mtDNA region is more abundant in total DNA preparations than in purified mitochondria (Fig. 1B). These data indicate that purification of mitochondria by sucrose gradient centrifugation appears to enrich structural intact mitochondria while damaged, ‘aged’ mitochondria which may have released copper to the cytoplasm are lost.

**Figure 2:**
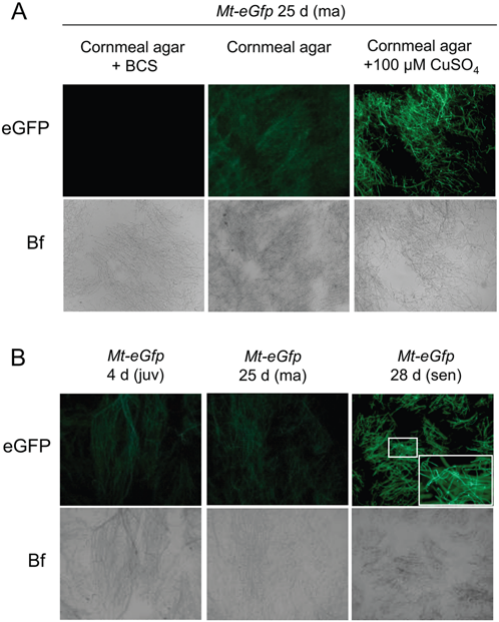
Copper-dependent activation of *eGfp* expression in the senescent state. (A) Transformant *Mt_eGfp* was grown on copper-deprived (BCS), standard and CuSO_4_-supplemented medium and subsequently analyzed by fluorescence microscopy. Below each panel the corresponding brightfield (Bf) pictures are shown. (B) Elevated *eGfp* expression in the senescent state of the *Mt_eGfp* transformant. The inset in the right-hand panel shows a magnification of the marked area. The eGFP protein is distributed within the cytoplasm.

In a second series of experiments we aimed at monitoring changes of cellular copper levels during ageing of *P. anserina in vivo* (Fig. 2). Towards this end, we constructed a reporter plasmid (pSM4-P*Mt*-EGFP) by cloning a 4.3 kbp promoter region of the copper inducible *PaMt1* gene upstream of an *eGfp* reporter gene. At high copper levels the *eGfp* gene of this plasmid should be expressed. Since the protein does not contain a mitochondrial target sequence it cannot enter mitochondria and remains in the cytoplasm. Protoplasts of the *P. anserina* wild-type (s) were transformed with the verified pSM4-P*Mt*-*eGfp* plasmid, and hygromycin B resistant transformants containing a complete genomic integration of the plasmid were selected by Southern blot analysis (data not shown). Copper regulation of the *PaMt1* promoter fragment was investigated by growing transformants on CuSO_4_-supplemented and copper-deprived (BCS/ascorbic acid) growth medium followed by microscopic analysis. Representative results of this analysis, exemplified for one transformant (mt-eGFP1), are shown in Fig. 2. As expected, fluorescence of a middle-aged *Mt_eGfp* transformant was found to increase with increasing amounts of copper in the growth medium (Fig. 2A).

Most importantly, fluorescence of cultures grown on standard corn meal medium is rather low in juvenile cultures and in cultures close to the senescent stage. Only senescent *Mt_eGfp* cultures display bright eGFP fluorescence (Fig. 2B) indicating that copper accumulation in the cytosolic fraction occurs late in the lifetime of *P. anserina*.

Taken together, the *in vitro* copper measurements and the *in vivo eGfp* expression studies both provide additional new evidence supporting the earlier findings which suggested an increase of cytosolic copper in senescent cultures of *P. anserina*.

### Release of a marker protein from the mitochondrial matrix in ageing cultures

**Figure 3:**
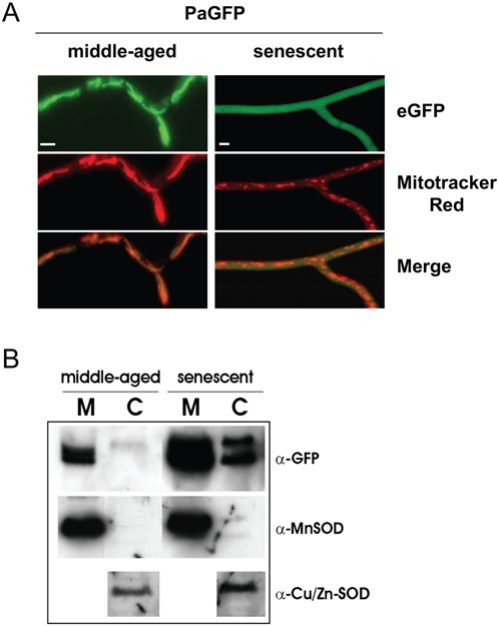
Contents of the mitochondrial matrix are released into the cytosol in senescent *P. anserina* strains. (A) The localization of eGFP directed into the matrix changes from mitochondrial in middle-aged strains (left) to cytoplasmic distribution in senescent strains (right). As control mitochondria are stained with MitoTracker® red CMXRos. Scale bar: 2 µm. (B) The microscopic results are verified by Western Blot analysis. eGFP (mature form and pre-protein) can be detected in the cytosol (C) of senescent strains (right), but not in middle-aged isolates (left) (M - mitochondria). Antibodies against the MnSOD (mitochondria) and the Cu/Zn-SOD (cytosol) were used as marker proteins for compartments and to show equal loading. The natively tetrameric MnSOD is retained within the mitochondria in senescence showing the size-limit for release should be between 27 kDa and ∼80 kDa.

In order to further support the hypothesis of an age-related release of components from mitochondria, we constructed an *eGfp* expression vector in which the *eGfp* open reading frame was fused to the mitochondrial target sequence (MTS) of the mitochondrial processing peptidase (MPP) from the closely related ascomycete *Neurospora crassa* [17]. Middle-aged cultures of the selected transformants expressing the introduced gene display tubular shaped fluorescent structures (Fig. 3A, left panel). Counterstaining with MitoTracker® CMXRos verified that the fluorescing structures represent mitochondria and demonstrate a mitochondrial localization of the eGFP marker protein. Strikingly, in hyphae of senescent cultures of the same transformant a diffuse green fluorescence occurs. MitoTracker® CMXRos staining identified distinct punctuate structures corresponding to fragmented mitochondria as they are characteristic for senescent *P. anserina* cultures [29] (Fig. 3A, right panel). These data suggest an age-related release of eGFP from mitochondria to the cytoplasm.

The results from the microscopic analysis were validated by Western blot analysis (Fig. 3B). In middle-aged transformants, eGFP was exclusively detected in the mitochondrial fraction (verified by mitochondrial MnSOD as a marker protein) and not in the cytoplasm. In contrast, in senescent cultures eGFP is located in both the mitochondrial and the cytosolic fraction. The two bands observed in the cytosolic fraction of the senescent culture correspond to the unprocessed pre-protein (upper band: eGFP plus MTS) and the mature form of the protein. The appearance of the mature form clearly shows that the detected eGFP has been within the matrix because removal of the MTS occurs only in this cellular compartment. Thus, the cytosolic eGFP-signal does not result from prevented import into but from release from mitochondria. Interestingly, in contrast to eGFP (27 kDa), the mitochondrial MnSOD (a functional tetramer of ∼80 kDa) was not relocated during ageing. These data suggest a size-dependent gated release mechanism as it is typically operating as one of the first steps in cell death leading to apoptosis or necrosis in mammals via ‘mitochondrial permeability transition’ (mPT) [30] and not to an uncontrolled general breakage of mitochondria.

### Targeting of a metallothionein to the mitochondrial matrix affects the accessibility of copper and lifespan

**Figure 4:**
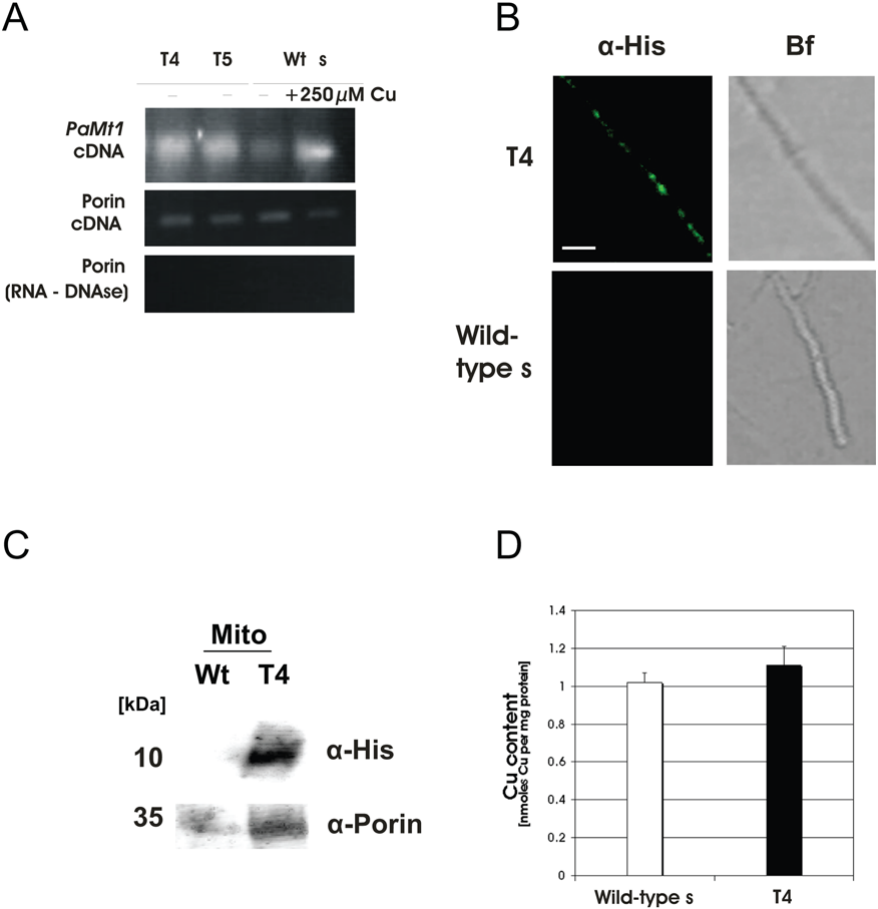
The copper-binding metallothionein PaMT1 is successfully targeted into the mitochondrial matrix. (A) The expression-level in the transformants T4 and T5, both express PaMT1 directed into the matrix, is comparable to wild-type s grown under pronounced copper-stress (+250 µM CuSO_4_) (upper panel). Porin was used as control for equal loading (middle panel) and absence of genomic DNA in RNA samples (lower panel). (B) *In situ* immunodetection of PaMT1-His verifies its mitochondrial localization in T4 (Bf: brightfield images) Scale bar: 10 µm. (C) Western blot analysis shows that his-tagged PaMT1 in transformant T4 is targeted to mitochondria. α-Porin: mitochondrial loading control. (D) Total copper content of mitochondria is unaffected in the matrix-PaMT1 transformant T4 (filled bar) compared to wild-type s (open bar) as measured by TXRF.

To elucidate the impact of mitochondrial copper on ageing and lifespan control more closely, we investigated the effect of reducing the accessibility of mitochondrial matrix copper. Toward this goal we constructed a plasmid in which the metallothionein gene of *P. anserina* [5] is fused to the mitochondrial target sequence (MTS) of the *Mpp* gene of *N. crassa*. The resulting plasmid was introduced into protoplasts of the *P. anserina* wild-type strain (s). Transcription of the metallothionein gene was demonstrated by semi-quantitative RT-PCR (Fig. 4A). The two transformants (T4, T5) grown on non-inducing medium contain transcript levels that are almost as high as in the wild-type strain grown on medium containing 250 µM CuSO_4_ as an inducer of the transcription of the endogenous metallothionein gene. To verify that PaMT1-his is indeed targeted to mitochondria, an *in situ* immunodetection was performed using α-His antibodies (Fig. 4B). Whereas the wild-type strain shows no signals, structures that resemble aggregated mitochondria are visible in mutants T4 and T5 (results shown for T4, Fig. 4B). Due to the necessary fixation for *in situ* immunodetection, the filamentous morphology normally observed for intact *P. anserina* mitochondria is lost. Western-blot analysis of mitochondrial proteins further proves the mitochondrial localization of PaMT1-his in the transgenic strains (results shown for T4, Fig. 4C). After validating the mitochondrial localization of PaMT1-his in T4 and T5, copper levels in mitochondria of these transformants were determined (Fig. 4D). Compared to the wild-type, they were not affected in the mutant strains (results shown for T4, Fig. 4D).

**Figure 5:**
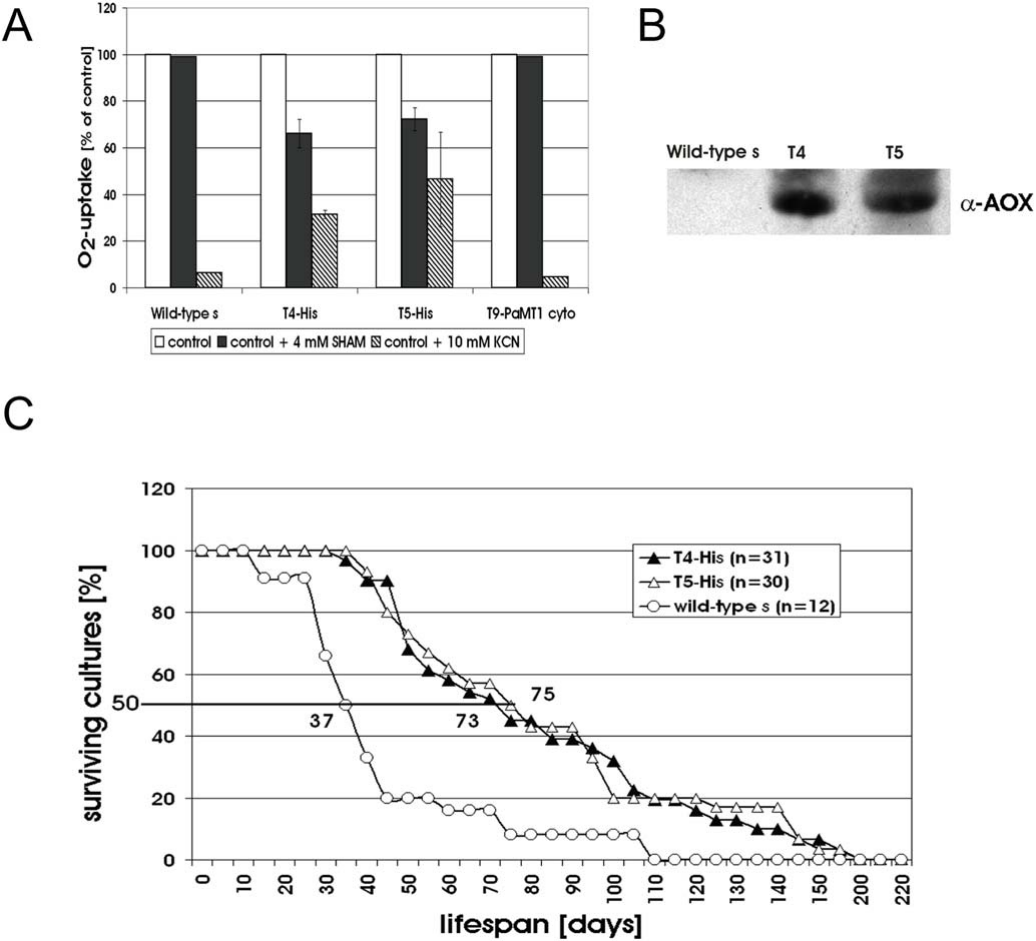
Targeting of PaMT1-his to mitochondria influences respiration and lifespan in *P. anserina*. (A) Cu-binding of PaMT1 reduces COX activity and induces AOX. Oxygen-consumption measurements of wild-type, two independent PaMT1 transformants (T4 and T5) and a control, overexpressing PaMT1 in the cytosol (T9-PaMT1 cyto) are shown. The percentage of respiration via COX or AOX, respectively, was determined by inhibition with 10 mM KCN or 4 mM SHAM, respectively. The values represent mean±SE. (B) Western blot analysis of isolated mitochondria of wild-type and the two PaMT1 transformants T4 and T5 probed with an antibody against the AOX of *S. guttatum*. (C) The mean lifespan of PaMT1 transformants T4 (n = 31) and T5 (n = 30) is doubled compared to the wild-type s (n = 12) (73 d and 75 d vs. 37 d) as determined in race tubes on standard cornmeal medium.

To assess physiological effects of targeting PaMT1 into the mitochondrial matrix of *P. anserina*, the type of respiration was determined. Oxygen-consumption revealed a reduction by 30% of KCN-sensitive COX-dependent respiration and a concomitant induction of SHAM-sensitive respiration via AOX in the transformants (Fig. 5A), compared to the wild-type which almost exclusively respires via COX. The induction of the AOX was also verified by Western blot analysis (Fig. 5B). Overall, the observed changes in copper-dependent respiration appear not to be due to a reduced amount of copper but rather result from a binding of copper by the mitochondrial targeted metallothionein. Presumably, this binding makes part of the mitochondrial copper pool unavailable for incorporation into COX.

Since induction of the alternative respiratory pathway is known to extend lifespan in *P. anserina* [7], [16], [31], we determined the lifespan of meiotic offspring of two independent transformants (T4, T5) on corn meal medium. On this medium, both transformants displayed a comparable lifespan of 73 and 75 days, respectively. Compared to the wild-type, this is a lifespan extension of about 100% (Fig. 5C).

### Impact of copper on the senescence of human diploid fibroblasts

After the demonstration of changes in cytoplasmic copper levels in *P. anserina* we asked whether this is specific for ageing in this fungal model system or whether such molecular changes are more general and conserved among organisms. To address this question we first investigated the impact of copper on the expression of three human copper-regulated genes and on senescence of human cells in culture.

**Figure 6:**
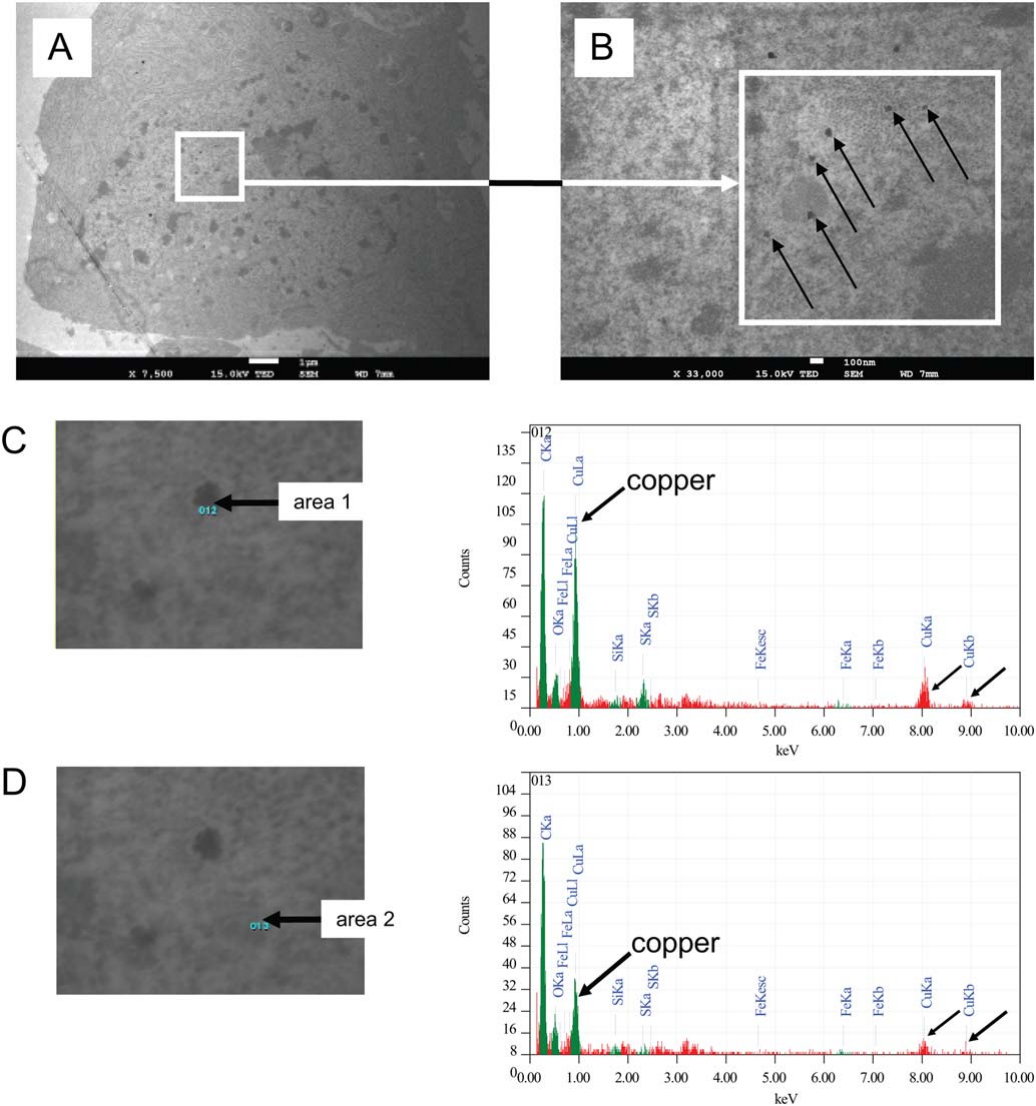
Detection of copper in WI-38 human diploid fibroblasts (HDFs) incubated with copper sulfate (CuSO_4_). Cells were incubated with 500 µM of CuSO_4_ for 16 h and were fixed just thereafter. Cells were treated for detection of copper by electron microscopy and Energy Dispersive X-ray (EDX) analysis. (A) Micrograph of a fibroblast incubated with CuSO_4_, magnification 7,500×. (B) Micrograph of a fibroblast incubated with CuSO_4_, magnification 33,000×. (C) Area selected inside a granule within a fibroblast incubated with CuSO_4_ and results of EDX analysis. (D) Area selected outside a granule within a fibroblast incubated with CuSO_4_ and results of EDX analysis.

We developed a model of sublethal exposure of human cells to copper. WI-38 fetal lung human diploid fibroblasts (HDFs) were incubated with 500 µM of copper sulfate (CuSO_4_) or sodium sulfate (Na_2_SO_4_) for 16 h. After the incubation, we detected copper inside cells by electron microscopy and Energy Dispersive X-ray (EDX) analysis (Fig. 6). We observed multiple granules inside the cells incubated with CuSO_4_. This was not seen in the cells incubated with Na_2_SO_4_ and in control cells. To detect copper, we selected an area inside a granule within fibroblasts incubated with CuSO_4_ and an area outside a granule, in the cytoplasm. The EDX analysis showed an increase of copper levels inside granules compared with cytoplasm. In this study, the reference chemical element is carbon and is present in the same order in the two areas. These data directly show that in our model system cellular copper levels increase by increasing copper in the culture medium.

**Figure 7:**
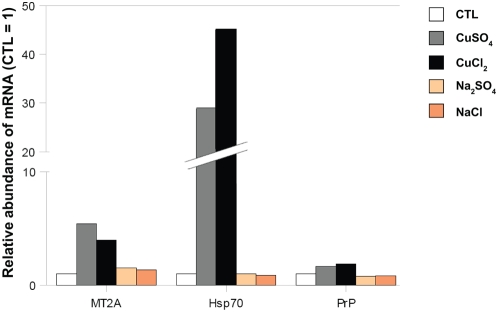
The abundance of mRNA of the copper-regulated genes (*MT2A*, *Hsp70* and *PrP*) is increased in WI-38 human diploid fibroblasts (HDFs) incubated with copper salts (CuSO_4_ or CuCl_2_) at 500 µM for 16 h. Sodium sulfate (Na_2_SO_4_) and sodium chloride (NaCl) were used as control salts. CTL represented the same treatment without copper or sodium salts. The results of real-time PCR are expressed as fold induction compared to controls (CTL). RPL13A, ribosomal protein L13A, was used as housekeeping gene. The presented graph is representative of two independent experiments.

Several human genes are known which are induced by copper in various human cell lines. Examples are genes encoding heat shock 70 kDa protein 1A (Hsp70/HspA1A) in HeLa cells [32], prion protein (PrP) in neurons [33] and metallothionein 2A (MT2A) in hepatoblastoma cells [34]. These genes can be induced by copper not only in human cells but also in species like yeast, fungi, invertebrates and several others [35]–[37]. To test if the cells used in our study react similarly to elevated copper levels in the culture medium, isolated RNA of WI-38 HDFs incubated with copper salts (CuSO_4_ or CuCl_2_) or with sodium salts (Na_2_SO_4_ or NaCl) was analyzed by real time RT-PCR (Fig. 7). The data were normalized to the transcription of the housekeeping gene coding for the ribosomal protein L13A (*RPL13A*). The transcript levels of these three copper-regulated genes were found to increase after the incubation with copper salts. The addition of sodium salts had no significant effect on the transcription on the genes of interest.

**Figure 8:**
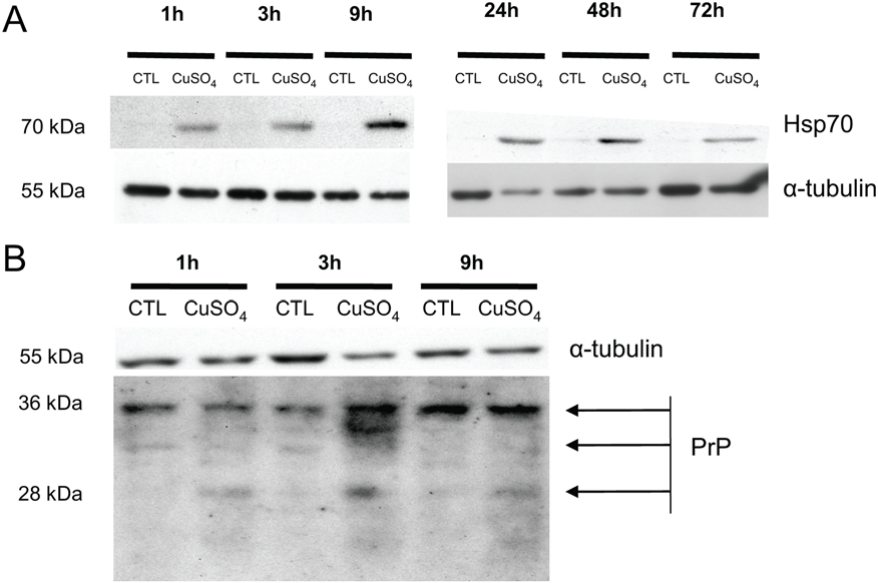
The abundance of the copper-regulated proteins (Hsp70 and PrP) is increased in WI-38 human diploid fibroblasts (HDFs) incubated with copper sulfate (CuSO_4_) at 500 µM for 16 h. Proteins were extracted at increasing times after the end of incubation with CuSO_4_. α-tubulin was used as a loading control. The presented blots are representative of two independent experiments.

We subsequently analyzed whether an increase of extracellular copper levels also affects the abundance of Hsp70, PrP and MT2A proteins. Proteins of WI-38 HDFs incubated with CuSO_4_ or controls cells were analyzed by Western blot method (Fig. 8). The abundance of Hsp70 increased in cells incubated with CuSO_4_ at 1, 3, 9, 24, 48 and 72 h after the end of incubation (Fig. 8A). For PrP, we observed another band at low molecular weight in cells incubated with CuSO_4_ at 1, 3, 9 h after the end of incubation (Fig. 8B). This band is likely to represent an unglycosylated PrP form. α-tubulin was used as a reference protein for equal loading on the gels. No antibody against MT2A worked in our experimental conditions. These results show that in WI-38 HDFs, the relative abundance of Hsp70 and PrP is increased in response to elevated copper levels.

**Figure 9:**
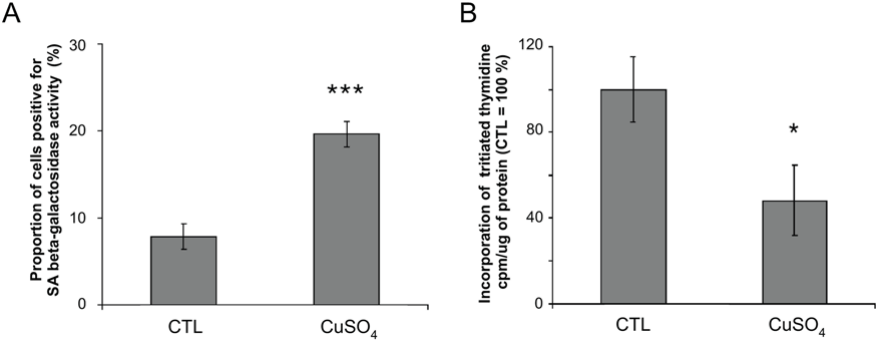
Analysis of senescence biomarkers in WI-38 human diploid fibroblasts (HDFs) incubated with copper sulfate (CuSO_4_) at 500 µM for 16 h. (A) Senescence-associated β-galactosidase. Control (CTL): cells not exposed to CuSO_4_ are considered as 100%. Mean value±SD of three independent experiments. Statistical analysis: Student's t-test. ***: p<0.001. (B) Estimation of the proliferative potential. At 48 h after the end of incubation, 10,000 cells were incubated for 24 h with 1 µCi [^3^H]-thymidine. Mean value±SD of three independent experiments. Statistical analysis: Student's t-test. *: 0.01<p<0.05.

Next we investigated whether biomarkers of senescence are induced by an increase of extracellular copper concentration (Fig. 9). As well known biomarkers of senescence the increase of the proportion of cells positive for senescence-associated β-galactosidase activity and growth arrest were analyzed. The results demonstrate an increase of the proportion of senescence-associated β-galactosidase positive cells (Fig. 9A) and a decrease of proliferative potential since the incorporation of [^3^H]-thymidine was strongly diminished (Fig. 9B) in WI-38 cells incubated with CuSO_4_.

### Age-related expression of copper-regulated genes in senescence of human diploid fibroblasts

**Figure 10:**
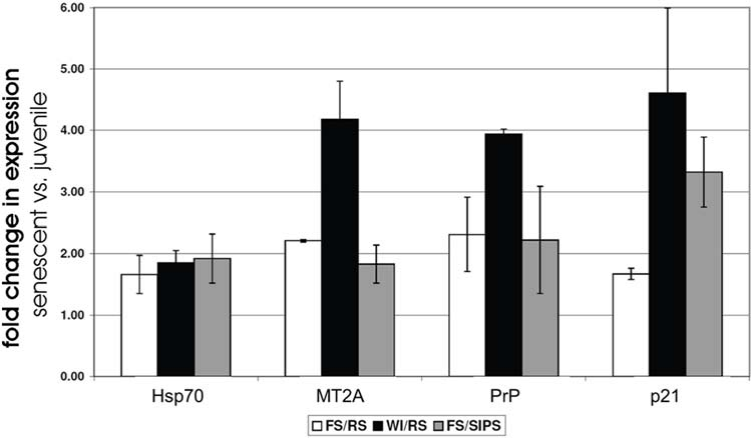
Overexpression of copper-regulated genes (*MT2A*, *Hsp70* and *PrP*) in senescent WI-38 and FS human diploid fibroblasts (HDFs). The results of real-time PCR are expressed as mean fold induction±SD in senescent vs. young cells. The expression of *GAPDH* was used as reference housekeeping gene. *p21* ^Waf-1^ (p21) was used as biomarker of senescence. The expression of these genes was investigated in three different strains of cells: FS/RS: replicatively senescent skin HDFs (white columns); WI/RS: replicatively senescent lung WI-38 HDFs (black columns); FS/SIPS: skin HDFs in stress-induced premature senescence (SIPS) via UVB treatment (grey columns).

Finally we investigated the age-related expression of the copper induced genes encoding Hsp70, PrP and MT2A. RNA of young and replicatively senescent cultures of different HDFs cell lines (BJ HDFs: skin fibroblasts; WI-38 HDFs: lung fibroblasts) grown in standard media. In addition, one cell line (BJ HDFs) in which senescence was induced prematurely by treatment with UV-B radiation [11] was analyzed. Transcription of *p21* ^Waf-1^ was used as a biomarker of senescence. In fact p21^Waf-1^ is a cyclin-dependent kinase inhibitor overexpressed in senescent cells [38]–[40]. The data were normalized to the transcription of the housekeeping gene coding for GAPDH. Transcript levels of these three copper-regulated genes were found to increase during senescence (Fig. 10). Depending on the cell lines and type of senescence mRNA abundance of Hsp70 increased by 1.66±0.31 fold and 1.92±0.40 fold. mRNA abundance of MT2A increased 1.83±0.31 fold and 4.18±0.62 fold, and mRNA abundance of PrP between 2.22±0.87 fold and 3.94±0.008 fold.

## Discussion

### Copper and ROS in P. anserina

The main goal of this study was to further investigate the role of copper in ageing of the fungal ageing model *P. anserina* and to translate the identified pathways to human cell cultures. Here we provide direct data strongly supporting the hypothesis that cytoplasmic copper levels are increasing in the senescent state. These findings identify a potential role of copper in the control of ageing in addition to the well established role of this metal in the mitochondrial respiratory chain and in ROS scavenging. While the induction of the copper-independent alternative oxidase (AOX) correlates with an extension in lifespan [1], [2], [7], [16] most likely due to the reduced ROS production by the AOX pathway [6], [7], the impact of the metal as a cofactor of SOD1 results in lowering ROS levels via the dismutation of the superoxide anion. In addition, changes in copper levels can effect gene expression and consequently modify specific functions. The molecular mechanism how increased copper levels affect gene expression can be the result of a direct interaction of copper with copper-sensing transcription factors like ACE1 and MAC1 in yeast [41], [42] and GRISEA in *P. anserina* [1], [4] giving rise to the induction or repression of a set of different genes. On the other hand, ROS generated by copper-mediated Fenton reaction may be involved in signalling and transcriptional regulation, as known for the metal-responsive transcription factor (MTF-1) in model systems from insects to mammals [43], [44].

### Copper and ROS in human cell lines

The three human genes investigated in this study are known to be transcriptionally induced by copper [33], [45], [46]. We also found that copper induces biomarkers of senescence such as an increase of senescence-associated β-galactosidase activity and growth arrest. It is known that an increase of abundance of β-galactosidase mRNA is responsible for the increase of senescence-associated β-galactosidase activity in ROS-induced senescence [47]. This increase is regulated by phosphorylation of p38^MAPK^ [48]. However, it is unclear whether copper induces transcription directly, e.g. via activation of a transcriptional activator, or indirectly by copper-mediated ROS [49], [50]. Support for the latter pathway comes from recent studies using HeLa cells in which mitochondrial ROS production was enhanced as the result of the inhibition of complex I by rotenone [51]. In these cells a drastic increase in metallothionein transcript and protein levels was observed [52] clearly suggesting a connection between ROS and metallothionein induction. Overexpression of *MT2A* was shown to ameliorate the effect of artificially induced ROS [52] indicating a protective role of metallothioneins against cellular damage and apoptotic death caused by oxidative stress [53]. The mode of activation by copper might also be independent of Fenton-reaction catalyzed ROS formation. This is suggested from studies with a mouse model for Wilson Disease, a human disease with chronic copper overload of the cells. In this model no activation of the ROS-scavenging system was detected during the copper accumulation and the onset of cellular damage [54]–[56].

### Putative origin of cytoplasmic copper in senescent cultures

One of the intriguing questions arising from this study is the source of additional copper in the ‘post-mitochondrial’ cell fraction. As suggested from previous investigations it is likely that the source of copper is the mitochondrion [2]–[4]. However, in this work we showed by applying TXRF measurements that copper content in sucrose purified mitochondria does not change during ageing in *P. anserina*. This unexpected result appears to be due to the fact that sucrose gradient purification of mitochondria may lead to the exclusion of the most severely damaged mitochondria from the analysis. Therefore only a set of mitochondria which are rather functional were analyzed while those which are severely impaired due to molecular damage or due to opening of the ‘mitochondrial permeability transition pore’ [30], [57], [58] may not band at the position where intact mitochondria are found. The soluble part of these mitochondria could therefore be part of the ‘sub-mitochondrial’ supernatant. This conclusion is supported by Southern blot analysis of the isolated mitochondria from senescent strains which show a rather high level of mtDNAs with no rearrangements in the investigated mtDNA region (Fig. 1B). Since mtDNA becomes strongly reorganized during ageing [22], [23], [28] the isolated mitochondria in fact do not appear to be the physiologically ‘oldest’ mitochondria isolated from the corresponding cultures.

### Putative mechanism of copper release from mitochondria

The molecular mechanism by which copper could be released from mitochondria is yet not finally elaborated. It is possible that there is a size-dependent gated release mechanism, as suggested by an age-related release of eGFP, a protein of 27 kDa targeted to the mitochondrial matrix, to the cytoplasm correlating with the change of mitochondrial morphology from filamentous to punctuate (this study). In contrast, MnSOD with a size of the functional tetramer of ∼80 kDa is not released from mitochondria. We suggest that copper in the mitochondrial matrix which, like in yeast, may be bound to a low molecular weight copper ligand [9] becomes released by a process resembling opening of a channel bridging both mitochondrial membranes in mammalian cells during apoptosis [59]. Previously, the opening of this ‘mitochondrial permeability transition pore’ (mPTP) was shown to lead to the release of small solutes like Ca^2+^ and components below 1.5 kDa [60]–[62] but also of proteins of higher molecular mass like adenylate kinase with a molecular weight of ∼30 kDa [63] or aspartate amino transferase with a molecular weight of ∼45 kDa [64]. The release of these proteins was found to be slower than the release of Ca^2+^ [63]–[67]. In mammalian cells, the opening of the mPTP occurs in early steps of apoptotic as well as necrotic cell death [30], [62], [68]. Significantly, recent studies revealed that the basic components of apoptotic machinery are encoded in the genome of *P. anserina* [69]. Interestingly, abundance of cyclophyllin D, which is supposed to be involved in mPTP opening, was found to increase in senescent cultures of *P. anserina* [70]. First experimental data suggesting apoptosis to occur also in the fungus linking this process to lifespan control and ageing have been reported recently [29], [69], [71]. Recent experiments indicate a role of mPTP opening in the final stage of senescence (manuscript in preparation). The opening of the mPTP during ageing may thus be responsible for the release of ligand bound copper from the mitochondrial matrix. Upon release copper would subsequently exert its impact on different molecular pathways leading to senescence and finally to cell death.

### Relocalization of copper in plants

An age-related relocalization of cellular copper, as it is reported in this study to occur during ageing of *P. anserina* was previously also reported to occur during leave senescence in higher plants [72]. Transcript levels of the post-Golgi Cu-transporter RAN1, a homologue of yeast CCC2, human WD and MNK [72], and of the Cu-chaperone CCH1, the homologue of ATX1 in yeast, delivering copper to CCC2 [73] were found to be increased in senescent leaves. The same was demonstrated for the genes coding for the ‘vegetative storage protein’ VSP2 [74] and several metallothionein (MT) isoforms [75]. The changes in transcript levels were suggested to result in the effective recycling of copper during leave senescence. According to this idea, copper is released to the cytoplasm after degradation of copper-containing proteins in the chloroplasts leading to the observed changes in the transcript levels of components of the copper homeostasis machinery [72]. The mobilized copper is subsequently transported into other plant organs where it is stored until needed for the development of new leaves in the next vegetation period.

The data from *P. anserina* presented here and in previous studies (e.g., [2], [5]) raise the question of whether chloroplasts are the only source of copper released to the cytoplasm during leave senescence. The recent demonstration of an unexpected high amount of freely accessible, non-proteinaceous copper within the mitochondrial matrix of *S. cerevisiae* and mice [8], [9], [76], [77] makes it very likely that mitochondria also serve as a copper reservoir in plants.

### Mitochondria as a cellular copper reservoir

In this study we provide data suggesting that the normal function of the mitochondrial matrix reservoir of copper is to serve as source for copper incorporation into COX during the assembly of this respiratory complex. In agreement with data obtained from *S. cerevisiae* [9] this conclusion is drawn from the observation that the metallothionein PaMT1 directed into the mitochondrial matrix competes with COX for copper and leads to a reduction of the amount of copper available for incorporation into subunits I and II of COX. In transformants containing PaMT1 in the mitochondrial matrix, COX activity was found to be reduced by ∼30% and a concomitant induction of the iron-dependent AOX was observed. In yeast, such an effect was only found in a mutant strain in which cellular copper is depleted due to deletion of the high affinity copper transporter CTR1 [9]. The targeting of PaMT1 into the mitochondrial matrix of *P. anserina* was found to lead to a doubling of the lifespan which partially can be attributed to a reduced generation of ROS in mitochondria respiring via the alternative pathway [6]. However, this reduced generation of ROS as toxic compounds may not be the only reason for the pronounced effect on lifespan. Here it is noteworthy to mention that the copper-depletion mutant grisea in which ∼80% of the electrons in the electron transport chain flow via the AOX [6] displays an increase in lifespan of only ∼60% [1], [2] in comparison to about 100% of the newly constructed transformants described in this paper. This difference may result from the tight binding of matrix copper to PaMT1 and a reduced level of accessible copper (e.g. bound to a ligand) that may serve as a signal after release into the cytoplasm for the induction of cell death. If this holds true, the time between opening of the mPTP (see above) and cell death may be prolonged in the matrix-PaMT1 mutants. Future experiments will investigate this possibility.

### Conclusions and outlook

Taken together, the data reported in this work strongly support an age-related increase of cellular copper and a novel role of this metal as a signal in the machinery controlling ageing and senescence, conserved from lower to higher eukaryotes. Further work will need to focus on the mode of signal transduction and the particular changes in cell physiology upon cytoplasmic copper increase. In this context the elucidation of the complete “copper transcriptome” in senescent stages compared to earlier lifespan stages appears to be important. The corresponding gene products may be suitable biomarkers of senescence and may lead to the identification of novel, yet unknown molecular pathways relevant to ageing. One good example supporting this expectation is the elucidation of the role of mitochondrial dynamics on ageing which was concluded from the identification of *PaDnm1*, a gene upregulated in the long-lived copper-uptake mutant grisea of *P. anserina* [29]. This gene is part of a pathway controlling mitochondrial fission. It was shown that mitochondrial dynamics and the structure of mitochondria play an important role for ageing not only in *P. anserina* but also in yeast [29].
